# Anisotropically Shaped Magnetic/Plasmonic Nanocomposites for Information Encryption and Magnetic-Field-Direction Sensing

**DOI:** 10.1155/2018/7527825

**Published:** 2018-08-30

**Authors:** Xiaojing Wang, Ji Feng, Huakang Yu, Yue Jin, Andrew Davidson, Zhiyuan Li, Yadong Yin

**Affiliations:** ^1^Department of Chemistry, University of California, Riverside, CA 92521, USA; ^2^Materials Science and Engineering Program, University of California, Riverside, CA 92521, USA; ^3^School of Physics and Optoelectronics, South China University of Technology, Guangzhou 510641, China

## Abstract

Instantaneous control over the orientation of anisotropically shaped plasmonic nanostructures allows for selective excitation of plasmon modes and enables dynamic tuning of the plasmonic properties. Herein we report the synthesis of rod-shaped magnetic/plasmonic core-shell nanocomposite particles and demonstrate the active tuning of their optical property by manipulating their orientation using an external magnetic field. We further design and construct an IR-photoelectric coupling system, which generates an output voltage depending on the extinction property of the measured nanocomposite sample. We employ the device to demonstrate that the nanocomposite particles can serve as units for information encryption when immobilized in a polymer film and additionally when dispersed in solution can be employed as a new type of magnetic-field-direction sensor.

## 1. Introduction

It is widely known that the plasmonic properties of metal nanostructures are mainly determined by their size, shape, and geometrical arrangement [1–4]. Compared to isotropic spherical particles, anisotropic nanostructures like rods and plates can offer greater tuning of plasmonic properties by controlling their shapes [5, 6] and orientations [7–9]. Instantaneous tuning of the orientation of the plasmonic nanostructures is crucial for the selective excitation and quenching of specific plasmon modes. Thus, the plasmonic properties can be dynamically controlled. For anisotropic plasmonic structures, such as gold nanorods (AuNRs) and nanoplates, the plasmon excitation peak can be easily redshifted into the near-infrared or infrared (NIR/IR) range by adjusting the geometric aspect ratio [1, 2, 5]. The advantage for shifting the plasmon excitation peak into the NIR/IR region mainly ties into sensor applications where there is little to no perceivable change in the visible spectrum. For example, for data encryption, information can be hidden to the human eye and the sensor's performance will not be easily influenced by the visible light pollution.

Among all the physical and chemical stimuli, the magnetic field has attracted a lot of research interest because it can alter the behavior of magnetic materials in a noninvasive, contactless manner [10]. Advantages of using an external magnetic field to control a chemical or physical system include nearly instantaneous response time, the directionality of the magnetic field vector, and low sensitivity to variations in experimental conditions [3, 10, 11]. The fast response and directionality are of particular importance when using magnetic materials to control the extinction values of anisotropic plasmonic nanostructures. We have successfully demonstrated that the orientation of gold nanorods could be dynamically controlled by binding them parallelly to the surface of superparamagnetic iron oxide nanorods. When applying an external magnetic field, the angle between the field direction and the direction of polarization of the incident light could be readily adjusted, thus resulting in the selective excitation of either transverse or longitudinal mode of the gold nanorods [3]. This proof-of-concept demonstration offers us numerous opportunities to develop functional devices based on the magnetically controlled plasmonic property.

When dispersed in solution, the plasmonic properties of the anisotropic nanostructures can be dynamically tuned or switched on and off using an applied magnetic field. The correlation between the magnetic field direction and the corresponding plasmonic extinction value can serve as the primary mechanism for design and fabrication of a new type of magnetic-field-direction sensor [12–14]. Recently, the inkjet-printed 1D arrays of Fe_3_O_4_ magnetic nanoparticles have been demonstrated with highly anisotropic magnetization and possess the ability for magnetic field sensing [15]. Also, for a fixed pattern of materials with the plasmonic property such as photonic crystals, it could be used for anticounterfeiting [16] or hiding information [17]. Likewise, when dispersed in a photocurable polymer, specific orientations of the nanostructures can be permanently fixed by UV polymerization [18, 19], and since nanostructures with different orientations exhibit unique extinction values, multiple fixed nanostructure arrays can be used for data encryption applications [20, 21]. The magnetic component should have an anisotropic shape, like the nanorod [22], to achieve the magnetic manipulation over the orientation of the plasmonic nanostructure.

Herein we report the successful synthesis of anisotropic magnetic/plasmonic Fe_3_O_4_ NRs@SiO_2_@Au core-shell nanocomposites and demonstrate magnetic control over the plasmonic properties in solution and fixed polymer films for magnetic-field-direction sensing and information encryption, respectively. Akaganéite (*β*-FeOOH) nanorods are used as the starting materials which are first coated with a layer of silica, reduced to magnetic state (magnetite), followed by a gold seed loading and seeded growth. The combination of the anisotropic magnetic nanorod and ellipsoidal gold nanoshell allows for magnetic control over the orientation of nanocomposites. We demonstrate that the angular dependence of the plasmonic extinction value can be adjusted with an external magnetic field and that nanocomposites arrays with select orientations can be fixed in a polymer film to preserve a given extinction value. Together with a homemade IR-photoelectric coupling device, the fabricated films can generate different output voltages dependent on their extinction values and therefore can be employed as the building blocks for information encryption. Moreover, based on the dynamic tuning of the plasmonic property in solution, we extend the working principle and fabricate a new type of magnetic-field-direction sensor and demonstrate the sensor performance by an actuator system.

## 2. Results

### 2.1. Synthesis of Anisotropic Magnetic/Plasmonic Nanocomposites

**Figure 1:**
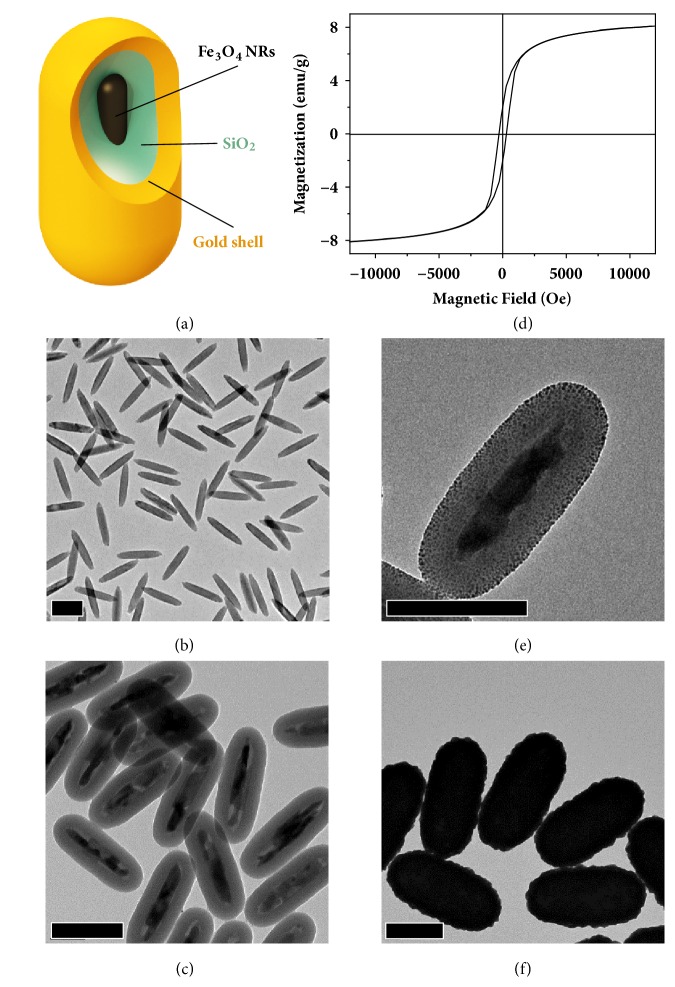
**Synthesis and characterization of anisotropic magnetic/plasmonic Fe**_3_ **O**_4_ **NRs@SiO**_2_ **@Au shell nanocomposites.** (**a**) Schematic illustration of the nanocomposite structure. (**b**) TEM image of *β*-FeOOH nanorods. The size of the nanorods is 286 ± 11 nm in length and 45 ± 3 nm in width. (**c**) TEM image of the magnetic Fe_3_O_4_ NRs@SiO_2_ after forming gas reduction. The thickness of the silica coating is 45 nm. (**d**) Magnetic hysteresis loop of Fe_3_O_4_ NRs@SiO_2_. (**e**) TEM image of the gold seed loaded NRs. (**f**) TEM image of Fe_3_O_4_ NRs@SiO_2_@Au shell after seeded growth to form a complete gold shell coating. The thickness of the gold shell is measured to be 36.5 nm. Scale bars in (**b**), (**c**), (**e**), and (**f**) are 200 nm.

There are two main challenges in the synthesis of anisotropic magnetic/plasmonic nanocomposite rods (Figure 1(a)). The first difficulty lies in the direct synthesis of high-quality magnetic iron oxide nanorods, with typical problems of limited dimensional control and particle aggregation [23, 24]. Indirect routes to magnetic nanorods employing a postreduction method have to be used [3, 18, 25, 26]. The second is that growing a uniform gold shell directly on bare iron oxide surface is considerably difficult through traditional wet chemistry techniques, while the growth of gold on silica surfaces has been reported in the literature [27]. For these reasons, we have developed a multistep synthetic route to the growth of gold nanoshells on magnetic nanorods to form the desired nanocomposites, which encompasses the following: (1) synthesis of *β*-FeOOH nanorods; (2) silica coating; (3) reduction of the *β*-FeOOH nanorods into magnetic Fe_3_O_4_ NRs@SiO_2_ by forming gas; (4) amine functionalization of the silica surface; (5) attachment and growth of gold seeds on the surface of nanorods to form the gold shell.

The synthesis of uniform *β*-FeOOH nanorods is accomplished by the hydrolysis of iron chloride. Figure 1(b) shows the transmission electron microscopy (TEM) image of the as-synthesized nanorods with an average size of 286 ± 11 nm in length and 45 ± 3 nm in width. The *β*-FeOOH nanorods are then modified with a layer of silica (45 nm) and reduced in a tubular furnace with a flow of forming gas to convert to magnetic Fe_3_O_4_. The silica coating has three functions. First, it serves as the protection layer to prevent the *β*-FeOOH nanorods from deforming during the reduction step. It also stabilizes the nanocomposites from aggregation induced by the magnetic dipole-dipole attraction after conversion to Fe_3_O_4_. Lastly, it serves as the substrate for further 3-aminopropyltriethoxysilane (APTES) modification and gold seed loading, acting as a bridge layer between the magnetic nanorod and the gold shell. Figure 1(c) shows the TEM image of the magnetic Fe_3_O_4_ NRs@SiO_2_. Due to the existence of the silica protection layer, the iron oxide core successfully maintains the rod structure. The size of the magnetic Fe_3_O_4_ NRs@SiO_2_ is measured to be 349 ± 8 nm in length and 139 ± 4 nm in width. The slight size shrinkage is due to the dehydration and deformation during the forming gas reduction. At the same time, the magnetic nanorods have very good dispensability in water. Before the forming gas reduction, the phase of the nanorods was determined to be akaganéite ([Sec supplementary-material-1], blue line), and the crystallinity is high. After reduction, the phase becomes magnetite ([Sec supplementary-material-1], red line). However, the crystallinity is poorer which we attribute to the structural deformation and shrinkage during the high-temperature treatment. Figure 1(d) shows the magnetic hysteresis loop of the Fe_3_O_4_ NRs@SiO_2_ with a magnetic saturation of 8.0 emu/g, which is relatively small, approximately one-tenth of the superparamagnetic colloidal nanocrystal clusters from our previous work [28]. The coercivity was measured to be 290 G ([Sec supplementary-material-1]), which confirms the ferrimagnetic property of the reduced samples. The weak magnetic saturation offers a benefit of avoiding particle aggregation in solution, which is often observed for ferrimagnetic particles, as the magnetic dipole-dipole interactions between the magnetic nanorods are sufficiently weak compared to the electrostatic repulsion between the silica shells. On the other hand, as discussed later, the magnetic saturation is high enough to allow effective control over the particle orientation using external magnetic fields.

For gold seed loading, the silica-coated magnetic nanorods are modified by APTES, offering abundant amino groups on the silica surface to bind the small gold seeds. An evenly distributed, high-density loading of gold seeds onto the nanorods, shown in Figure 1(e), facilitates the formation of gold shells with uniform thickness and coverage. The growth of the gold shell is conducted using a prepared growth solution as the gold precursor and formaldehyde as the reducing agent. During the growth of the gold shell, the extinction spectra initially gradually redshifts to 765 nm followed by the emergence of a new peak near 650 nm, becoming stronger as the gold shell thickness increases ([Sec supplementary-material-1]a). When only 1 mL of growth solution is added, the gold seeds only grow larger but do not form a continuous shell ([Sec supplementary-material-1]b). When 4 mL total of growth solution is added, the larger gold nanoparticles coalesce and cover most of the nanorod surface but still do not form a complete shell ([Sec supplementary-material-1]c). Finally, when adding a total of 12 mL growth solution, a continuous and complete shell is formed ([Sec supplementary-material-1]d). Figure 1(f) shows the TEM image of the as-synthesized Fe_3_O_4_ NRs@SiO_2_@Au shell nanocomposites grown using in total 18 mL of growth solution and having a gold shell thickness of 36.5 nm (Figures [Sec supplementary-material-1]e and [Sec supplementary-material-1]f for TEM images before and after removing the Fe_3_O_4_/SiO_2_ core). The key to the successful seeded growth is to avoid any self-nucleation events. To accomplish this, a fast reaction is preferred initially to avoid ripening of the gold seeds into large nanoparticles, which will cause the density of the seeds to decrease and limit the formation of a complete uniform shell. Furthermore, stabilizing the gold shell with additions of PVP (polyvinylpyrrolidone) is necessary throughout subsequent growth steps to prevent aggregation.

### 2.2. Magnetically Controlled Angle-Dependent Plasmonic Property

**Figure 2:**
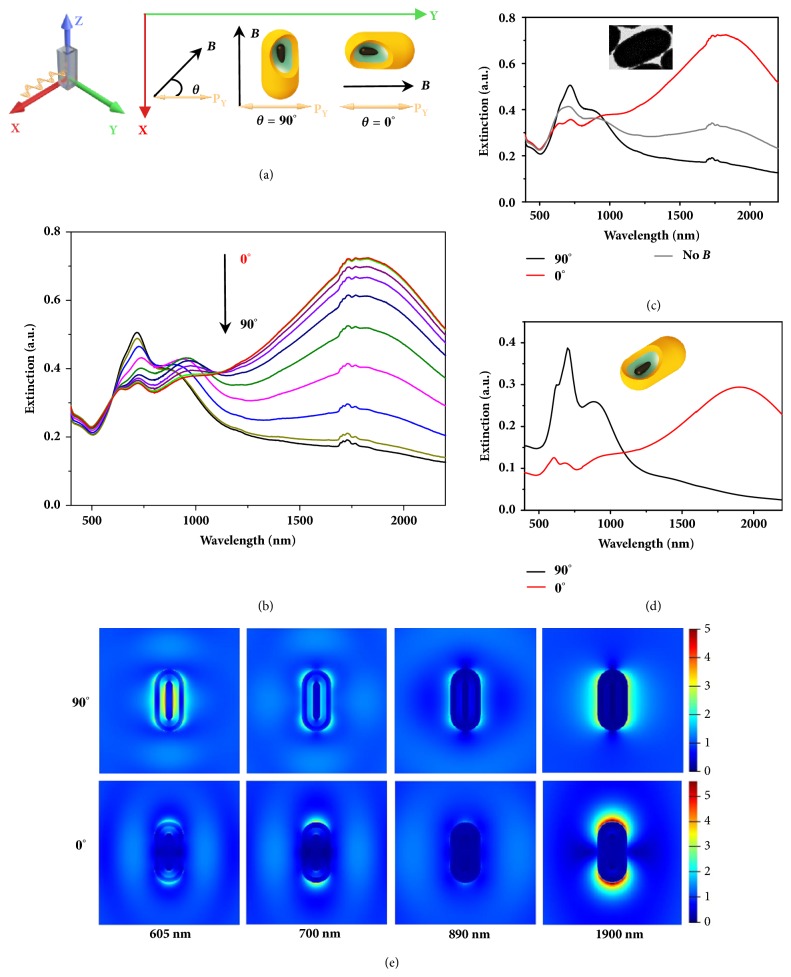
**Magnetically controlled angle-dependent plasmonic property of the nanocomposites.** (**a**) Schematic representation of the setup for plasmonic extinction measurement. (**b**) Extinction spectra measured when changing *θ* from 0° to 90° by 10° each time. The spectra are measured in a solution with CS_2_ as the solvent. (**c**) Extinction spectra of the nanocomposites with *θ* of 90 ° and 0° and without applying the external magnetic field, respectively. The inset shows the TEM image of a typical nanocomposite. (**d**) Calculated extinction spectra using the finite-difference time-domain (FDTD) method with *θ* of 90 ° and 0°. The inset shows the scheme of the nanocomposite used for simulation. (**e**) Electric field distributions, from left to right, under different excitation wavelengths of each peak as shown in extinction spectra: located at 605 nm, 700 nm, 890 nm, and 1900 nm, respectively. The top row is when incident light polarized along the transverse axis (*θ* = 90°), and the bottom row is when incident light polarized along the longitudinal axis (*θ* = 0°).

In the presence of an external magnetic field, the ferrimagnetic nanocomposites align parallel to the direction of the magnetic field to minimize their magnetic potential energy and dipole-dipole interaction [3, 18, 29]. Because of this, the angle-dependent plasmonic property due to the gold nanoshell component of the nanocomposites can be readily tuned by the magnetic field.  Figure 2(a) shows the schematic representation of the experimental setup for plasmonic extinction measurements. Linearly Y-polarized broadband light is incident along the X-axis. The orientation of nanocomposites is adjusted by applying an external magnetic field in the XY plane. The angle between the polarization direction of the incident light and the magnetic field is defined as *θ* (as schematically shown in Figure 2(a)). While *θ* is 0°, the polarization of excitation is only along the longitudinal axis of the nanocomposites; while *θ* is 90°, the polarization of excitation is then across the transverse axis of the nanocomposites. When varying *θ* from 0° to 90° by 10° each time, the extinction spectra gradually evolve from the red line to the black line as shown in Figure 2(b). The extinction peak near 1820 nm gradually decreases, while the peaks at shorter wavelengths, 640 nm, and 720 nm, gradually rise. All the extinction intensity changes are due to the orientation change of the gold shell alone. Any contributions from Fe_3_O_4_ NRs@SiO_2_ to the nanocomposite spectra are not observed under similar control experiments performed without the gold shell component ([Sec supplementary-material-1]). The small peaks located in the range of 1650–1800 nm are verified to be CS_2_ solvent employed in the measurement ([Sec supplementary-material-1]). In the absence of a magnetic field, the nanocomposites are randomly distributed in solution, and the measured extinction spectrum lies in between the red and black line, as shown in Figure 2(c) (grey line).

In the case of anisotropic plasmonic structures such as nanorods, two main surface plasmon resonance (SPR) modes exist, a longitudinal mode along with nanostructure's lengthwise axis and a transverse mode across the shorter width of a nanostructure. These SPR modes can be selectively excited by polarized light [5]; however, because the orientation of the nanocomposites can be dynamically controlled by an external magnetic field, the individual SPR modes can be selectively excited without changing the polarization of the incident light. By rotating the external magnetic field lines between *θ* = 90° and *θ* = 0°, the transverse or longitudinal SPRs can be excited, respectively. When 0° < *θ* < 90° the extinction spectra are characteristic of both SPR modes with relative magnitudes dependent on the angular orientation.

The finite-difference time-domain (FDTD) simulation method is used to investigate the relationship between the nanoshell structure and the plasmonic features of the extinction spectra. Simulated extinction spectra for *θ* = 90° and *θ* = 0° are presented in Figure 2(d) which are in good agreement with the experimental results. For *θ* = 90°, the transverse SPR is the primary excitation with extinction peaks in the visible wavelength range; for *θ* = 0°, the longitudinal SPR is primarily exited exhibiting a broad extinction peak across the near-infrared wavelength range.  Figure 2(e) depicts the electric field distributions located at 605 nm, 700 nm, 890 nm, and 1900 nm of both transverse and longitudinal SPR modes for the simulated nanocomposites. At 1900 nm, the electric field distributions due to the individual SPR modes are clearly defined on the outer surface regions of the gold shell. However, the shorter wavelength modes are more convoluted due to the hollow structure of the nanorod which can be seen by resonance modes within the shell.

For *θ* = 90°, at short wavelength, e.g., 890 nm, transverse surface plasmon resonance (TSPR) of outer shell becomes quadrupole, and plasmonic resonance of inner core (dipole mode) becomes visible, and the total plasmonic extinction becomes hybridized. Moreover, the quadrupole mode becomes even distinct when wavelength moves to even shorter, e.g., 700 nm (Figure 2(e), first row, second column). Moreover, the quadrupole mode is so strong that four lobes of radiation pattern can be observed. For shorter wavelength (e.g., 605 nm), the plasmonic resonance becomes more hybridized since the strength of plasmonic resonance of inner core turns out to be more significant. On the other hand, for *θ* = 0°, as the polarization of excitation now is along the longitudinal axis, longitudinal SPR modes are excited, as shown in Figure 2(e). For the wavelength of 1900 nm, it shows a very clean longitudinal dipole mode of the outer shell. At shorter wavelengths, high order longitudinal SPR modes are excited (e.g., 890 nm) while oscillation of the inner core becomes more and more evident, and total plasmonic resonance is thus hybridized. Besides, extinction coefficients of longitudinal SPR spectra (i.e., *θ* is 0°) are more significant than that of transverse ones, which is quite consistent with the measured extinction spectrum. Overall, simulated extinction spectra are in good agreement with experimental data, except for slightly peak intensity change and peak position shift, which is possibly due to the imperfect nature of the synthesized gold shell.

### 2.3. Nanocomposite Array for Information Encryption

**Figure 3:**
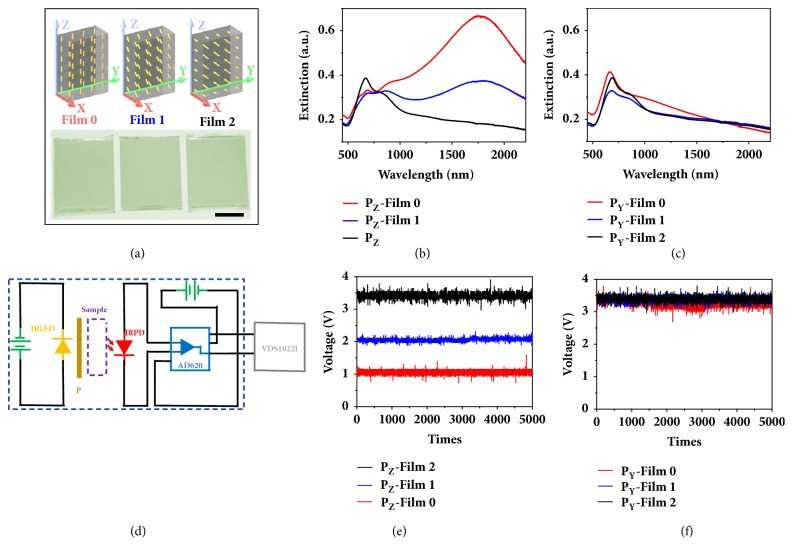
**The electric response of the nanocomposites array when tested by a homemade IR-photoelectric coupling system.** (**a**) Schematic illustration of the three polymer films consists of nanocomposites with different orientations (top row) and the digital images (bottom) of the fabricated film. Scale bar is 1.0 cm. (**b, c**) Extinction spectra of the film measured under the illumination of linearly Z-polarized light (**b**) and linearly Y-polarized light (**c**). (**d**) Diagram of the homemade IR-photoelectric coupling system (IRPECS). (**e, f**) The voltage of the film measured under the illumination of linearly Z-polarized light (**e**) and linearly Y-polarized light (**f**).

The combination of plasmonic and magnetic properties make these nanocomposites very promising for a wide range of applications, especially in areas where dynamic tuning of the plasmonic property is required. By taking advantage of the effective orientational control of the nanocomposites, here we first demonstrate an application of the nanocomposites for information encryption by fixing the aligned nanocomposites inside polymer films.  Figure 3(a) shows the schematic illustration for the fixation of the nanocomposites inside a poly(ethylene glycol) diacrylate (PEGDA) film. The nanocomposite film arrays are fixed in the XZ plane, and the angle between the longitudinal axis of the nanocomposites and Z axis is set to 0°, 45°, and 90° to form films** 0**,** 1**, and** 2**, respectively. The bottom image shows that the three films have a very similar appearance and is difficult to distinguish between them with the naked eye. The extinction spectra of the films using both linearly Z-polarized and Y-polarized light are shown in Figure 3(b). When the incident light is polarized along the Z axis, Film** 0** shows the highest extinction, as *θ* = 0°, Film** 2** shows the lowest extinction, as *θ* = 90°, and Film** 1** shows that where *θ* = 45°, the extinction lies in between that of Film** 0** and Film** 2**. It is worth noting that the extinction difference is primarily in the NIR range and there is only a slight difference in the visible range which explains why it is difficult to distinguish the three films with our human eyes. In the case of the Y-polarized light, for all the three films *θ* = 90° as all the nanocomposites are aligned in the XZ plane, and the extinction spectra are nearly the same, as shown in Figure 3(c), since no contribution from the longitudinal mode is exhibited.

Based on the selective extinction property of the nanocomposite films, we designed a homemade IR-photoelectric coupling system (IRPECS).  Figure 3(d) shows the diagram of the IRPECS. An IR-LED having a broad emission peak (full width at half maximum of ≈150 nm and peak intensity near 1570 nm; [Sec supplementary-material-1]a) is used as the incident light source in combination with a polarizer to select the linearly polarized light. An IR photodiode ([Sec supplementary-material-1]b) together with a signal amplifier is used to detect the extinction intensity of a sample film and output a corresponding voltage signal. The output voltages of repeated scans (at every 1 ms) for Film** 0**, Film** 1**, and Film** 2** with both Z-polarized and Y-polarized light are shown in Figures 3(e)-3(f). It can be seen that the voltage signal is inversely proportionate to the extinction value. When the films are measured with Z-polarized light, Film** 0**, Film** 1**, and Film** 2** have output voltages of 1.076 V, 2.058 V, and 3.406 V, respectively. When Y-polarized light is used, all films show similar output voltages ranging from 3.255 V to 3.395 V as they have very similar extinction values.

**Figure 4:**
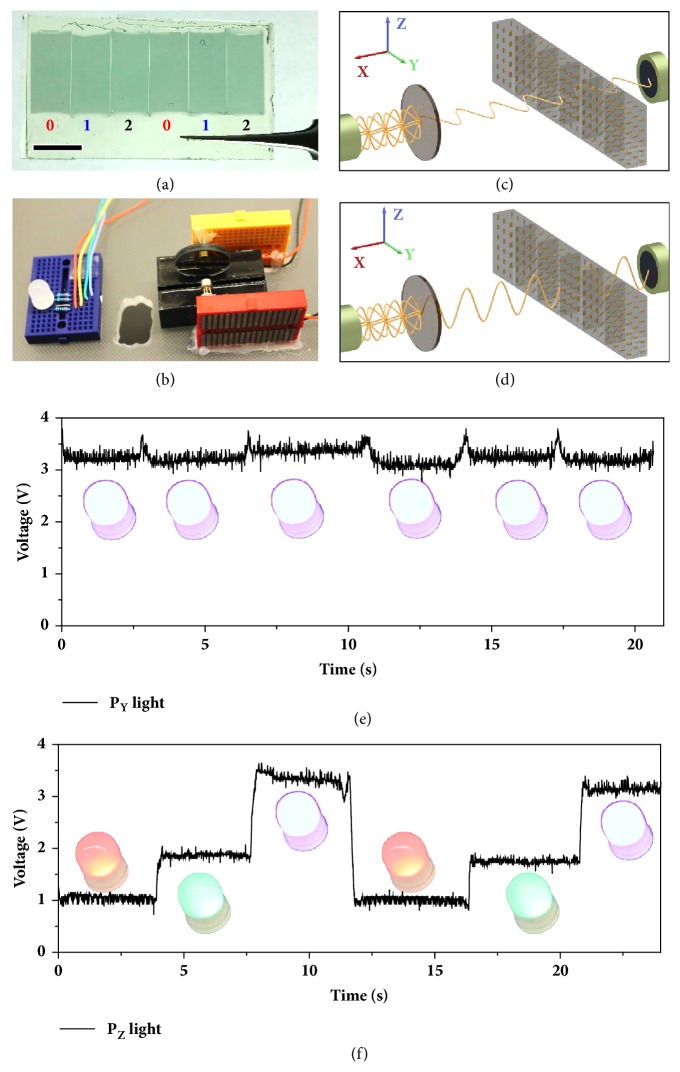
**Nanocomposites array for information encryption.** (**a**) Digital image of the fabricated film which consists of six building blocks. The sequence of the six columns is film** 0**,** 1**,** 2**,** 0**,** 1**,** 2**. Scale bar is 1.0 cm. (**b**) Digital image showing the IR light source, sample stage and photodiode part of the homemade IR-photoelectric coupling system (on the right), and a common anode LED which connected to the system through ARDUINO (on the left). (**c, d**) Scheme showing the two decryption methods for the fabricated polymer film when using linearly Y-polarized light (**c**) and Z-polarized light (**d**), respectively. (**e, f**) Different voltage readings of the film when measured under the illumination of linearly Y-polarized light (**e**) and Z-polarized light (**f**). Insets: corresponding LED color when reading the specific column of the film.

To further demonstrate the use of the nanocomposites for information encryption, a six-column film array comprised of films having similar orientations to Film** 0**, Film** 1**, and Film** 2**, (labeled** 0**,** 1**,** 2**) is constructed.  Figure 4(a) shows the digital image of the nanocomposites film array with six building blocks having a sequence of** 0**,** 1**,** 2**,** 0**,** 1**,** 2**. The hidden information encrypted in the film can be read out using the IRPECS, with the sample measurement portion of the system shown in Figure 4(b). To simplify the readout for the user, the IRPECS is combined with an identification system (also shown in Figure 4(b)) having an RGB-LED in which the color shown indicates the encrypted pattern. Briefly, the output voltage for the tested film that comes from the IRPECS was used as an analog input signal for programmed ARDUINO, which controls the emission color of the LED light. [Sec supplementary-material-1] shows the diagram of both the IRPECS and identification system. For encryption patterns** 0**,** 1,** and** 2**, the emission colors are red, green, and purple (red + blue), respectively. Shown in Figure 4(e), the LED readout shows the same purple color for all six subunits of the encrypted film array when measured using Y-polarized light ([Sec supplementary-material-1]). When the Z-polarized light is used, the three subunits of the film array cause the LED to vary in color among purple, green, and red (Figure 4(f) and [Sec supplementary-material-1]). Based on the LED color and a decryption key, the readout can be recorded as** 012012**.

The complexity of the encryption system developed here can be greatly expanded by increasing the number of combinations and subunits used in a film array. For example, assuming the same number of subunits** 0, 1, 2**, a nanocomposite film array with N columns will have 3^N^ different combinations. When N = 3, there are 27 combinations ([Sec supplementary-material-1] for the scheme and [Sec supplementary-material-1] for a dynamic changing video). For the film that we fabricated in this paper where N = 6, the total combinations will be 3^6^ = 729. Furthermore, the voltage outputs can be separated into additional discrete subunits dependent on the sensitivity of the detection system used; herein we simply use 3, but there remains room between those three voltages to utilize additional orientations of the nanocomposites and their corresponding extinction values. For example, if we add two additional nanocomposites films with orientations of *θ* = 22.5° and *θ* = 67.5°, then there would be five total different subunits. With a six-column film array, the total combinations can exponentially increase to 5^6^ = 15625.

Because of the large extinction value range of the magnetic/plasmonic nanocomposites, the complexity of the encryption system can be increased to the limits of the detection system. Additionally, the system meets a typical advantage of NIR encryption being indistinguishable to the human eye expanding its usefulness in other applications such as anticounterfeiting. A distinct advantage of this system over others is that the information hidden in the film can only be read with the correct polarization of light, providing improved security of the encrypted information. The nanocomposites developed can be used for even higher level encryption than exhibited here based on their magnetically tunable NIR plasmonic extinction properties.

### 2.4. Nanocomposite Dispersion for Magnetic-Field-Direction Sensing

Magnetic field sensing involves a variety of magnetic sensors to detect the presence, strength, or direction of magnetic fields. Most existing magnetic sensors are fabricated based on the Hall effect or giant magnetoresistance [12, 13]. However, in our design, we utilize the angle-dependent plasmonic property of the nanocomposites. When dispersed in solution, the orientation of the nanocomposites aligns parallel to external magnetic field lines. Employing the IRPECS and observing the output voltage, the direction of the external magnetic field can be deduced. This concept is the foundation of a new type of magnetic-field-direction sensor.

**Figure 5:**
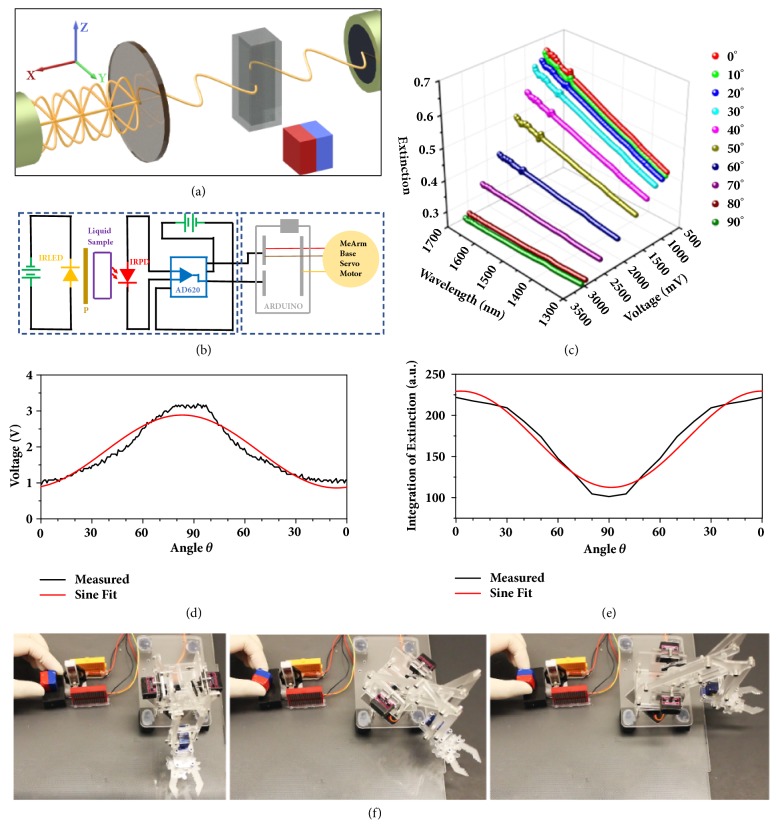
**The magnetically controlled real-time photoelectric response of the nanocomposites in solution and its application for magnetic-field-direction sensor.** (**a**) Schematic representation of the sample stage part. A cuvette containing the sample solution was placed in between the light source and the photodiode. A permanent magnet was used to dynamically control the orientation of the nanocomposites in XY plane. (**b**) Circuit diagram of the magnetic-field-direction sensor (left part) and an actuator (right part). (**c**) Extinction spectra and photoelectric response of the nanocomposites in solution when changing *θ* from 90 ° to 0°, by 10° each time. (**d**) Voltage plotted over *θ* angle and its sine fit. (**e**) Integration of the measured extinction from 1300 nm to 1700 nm of the nanocomposites in PEGDA solution plotted over *θ* angle and its sine fit. (**f**) Digital images showing the magnetic-field-direction sensor based actuator system. From left to right, the robotic arm moved accordingly when changing the direction of the external magnetic field.

Figure 5(a) shows the scheme of the sensor where the cuvette contains the liquid suspension of nanocomposites. Upon changing the external magnetic field direction in the XY plane, the output voltage generated by the IRPECS changes accordingly. The complete system diagram is shown in Figure 5(b). We measured the extinction spectra and the output voltages of the same liquid sample when changing *θ* from 90° to 0° by 10° each time. As plotted in Figure 5(c), from 1300 nm to 1700 nm, the extinction gradually increased while *θ* changes from 90° to 0°, but the corresponding output voltages gradually decreased. The correlation between *θ* and the output voltage was further determined by a quasicontinuous measurement. A servo motor equipped with a permanent magnet provides the external magnetic field, and the motor was programmed to rotate continuously at a speed of 10° per 50 ms ([Sec supplementary-material-1]a and [Sec supplementary-material-1]). The output voltages are plotted over time ([Sec supplementary-material-1]b) and show very consistent results over six measurement cycles. The output voltages are plotted against angle *θ*. As shown in Figure 5(d), the voltage output resembles a sine function which is fit by *V*_*output*_ = 1.873 + 1.103sin⁡[*π*(*θ* − 37.56)/90.96]. The sine fit is in the form of *y* − *y*_0_ + Asin⁡[*π*(*x* − *x*_*c*_)/*ω*], where A is the amplitude of the peak, *ω* is the peak width, and *y*_0_ and *x*_*c*_ are constants. Since the output voltage is directly related to the extinction value of the nanocomposites suspension, the integration of extinction from 1300 nm to 1700 nm is plotted against *θ*. Figure 5(e) shows a cosine fit, which explains why the output voltage is compliant with a sine fit since the extinction value is inversely proportionate to the output voltage.

For demonstration purposes towards a unique application of the direction sensor, we designed an electronic actuator system, which responds to the magnetic field direction. Figure 5(b) shows the schematic representation of the actuator system. When changing *θ* by manually rotating the permanent external magnet placed beside the cuvette, the robotic arm can respond and rotate accordingly (shown in Figure 5(f) and [Sec supplementary-material-1]). Here, we only confine the rotation of the robotic arm within 90°. However, it can be programmed to respond in a broader range, e.g., 180° or 360°, depending on the specific motor type and how to program it. It is worth noting that, for the particular sample that we tested, the minimum field strength required to drive the orientational change of the nanocomposites was determined to be 0.2 mT. Such a low detection limit allows a broad range of practical applications.

## 3. Discussion

We have successfully developed the synthesis of anisotropic magnetic/plasmonic Fe_3_O_4_ NRs@SiO_2_@Au nanocomposites by a postreduction and seeded growth method studied their responsive plasmonic properties and demonstrated their potential applications. By applying an external magnetic field, the orientation of the nanocomposites can be dynamically controlled, which allows the measurement of the angle-dependent plasmonic extinction properties of the as-synthesized materials. Furthermore, we demonstrate that the nanocomposites can be fixed in polymer films and serve as excellent building blocks for information encryption. We designed and fabricated a homemade IR-photoelectric coupling system to generate an output voltage based on the extinction magnitude of the measured sample. Taking advantage of the instantaneous tuning of the orientation of nanocomposites in solution, we have further developed a novel magnetic-field-direction sensor which can be employed to actuate a robotic system. It is anticipated that miniaturization and integration of functional devices based on anisotropic magnetic/plasmonic Fe_3_O_4_ NRs@SiO_2_@Au nanocomposites can benefit many other applications such as magnetic field mapping, precise magnetosensitive control and measurement, and optical computing.

## 4. Materials and Methods

### 4.1. Chemicals

Iron(III) chloride hexahydrate (FeCl_3_·6H_2_O), gold(III) chloride trihydrate (HAuCl_4_·3H_2_O), poly(acrylic acid) (PAA, average M.W. 1800), tetraethyl orthosilicate (TEOS), formaldehyde (HCHO), 3-aminopropyltriethoxysilane (APTES), tetrakis-(hydroxymethyl)phosphonium chloride (THPC), 1-octanethiol, poly(ethylene glycol) methyl ether thiol (PEG-SH), poly(ethylene glycol) diacrylate (PEGDA, average M_n_ 575), and phenylbis(2,4,6-trimethylbenzoyl)phosphine oxide (photoinitiator) were purchased from Sigma-Aldrich. NH_4_OH solution (28%), NaOH, K_2_CO_3_, and CS_2_ were purchased from Fisher Scientific. Ethanol (200 proof) is purchased from Decon Laboratories Inc. Polyvinylpyrrolidone (PVP, K12, average M.W. 3500) is purchased from Acros Organics. All the chemicals were used as received.

### 4.2. Synthesis of Magnetic Iron Oxide Nanorods (Fe_3_O_4_ NRs@SiO_2_)

Akaganéite (*β*-FeOOH) nanorods are synthesized by hydrolysis of iron chloride and used as the starting materials. In a typical synthesis, 400 mL of 0.1 M FeCl_3_*∙*6H_2_O solution is prepared in a sealed glass bottle. The solution is then put into an oven at 40°C and maintained at that temperature for 6 days. The solid product is collected by centrifugation (11000 rpm, 3 min) and washed with water several times. Next, 40 mL of the *β*-FeOOH nanorods (9 mg/mL) is stirred overnight with an equal volume of PAA (0.1 M, pH = 8-9 and adjusted by adding NH_4_OH solution) to facilitate silica coating on the nanorod surface. Excess PAA is removed by washing with water three times and finally dispersed in 36 mL water, and the silica coating is performed using a modified Stöber method as follows. *β*-FeOOH-PAA (12 mL, 10 mg/mL), NH_4_OH solution (28%, 4 mL), and ethanol (80 mL) are first mixed in an Erlenmeyer flask by vigorous stirring, followed by the addition of 200 *μ*L TEOS. After 30 min reaction, the *β*-FeOOH NRs@SiO_2_ nanorods are washed with water and collected by centrifugation (7500 rpm, 3 min) three times and dried in an oven at 60°C. Conversion of FeOOH NRs@SiO_2_ to Fe_3_O_4_ NRs@SiO_2_ magnetic nanorods is achieved by a forming gas reduction at 360°C for two hours in a tube furnace. After reduction, the magnetic nanorods are collected and dispersed in 40 mL ethanol.

### 4.3. The Growth of Gold Shell on Magnetic Nanorods

The growth of the gold shell on Fe_3_O_4_ NRs@SiO_2_ magnetic nanorods is performed following a modified gold seed loading and seeded growth method reported by Halas et al. [30]. Initially, the Fe_3_O_4_ NRs@SiO_2_ surface is amine modified by APTES. In a typical procedure, 40 mL of the prepared Fe_3_O_4_ NRs@SiO_2_ in ethanol (3 mg/mL) solution is transferred into a 100-mL three-neck flask and heated to 78°C at which time 200 *μ*L of APTES is added. The mixture is refluxed at 78°C for 3 hours under vigorous stirring and then allowed to continue stirring at room temperature overnight. The amine functionalized nanorods (Fe_3_O_4_ NRs@SiO_2_-APTES) are collected by centrifugation, washed three times with ethanol, an additional three times with water, and finally dispersed in 12 mL of water.

Gold seeds (1-2 nm) are synthesized following a reported protocol [31] with slight modification. Briefly, 96 mL of water, 315 *μ*L of 2.0 M NaOH, 126 *μ*L of 16 wt% THPC aqueous solution are mixed in an Erlenmeyer flask for 5 min at which time 420 *μ*L of 0.25 M HAuCl_4_·3H_2_O is quickly added. The color of the mixture undergoes a sudden change from colorless to dark brown. The as-synthesized gold seeds were further aged at 4°C for approximately one week before use. For gold seed loading, 1 mL of Fe_3_O_4_ NRs@SiO_2_-APTES (10 mg/mL) is added to 10 mL of the THPC gold seed solution drop-wise under sonication. After the addition, the mixture is stirred for 60 min. Excess unbound gold seeds are removed by washing with water twice, and the seed loaded nanorods (Fe_3_O_4_ NRs@SiO_2_-Au seed) are dispersed in 15 mL of water.

The growth of gold seeds into the gold shell is performed using formaldehyde as a reducing agent and a prepared gold growth solution [32]. Gold growth solution is prepared by mixing 49.6 mg K_2_CO_3_ with 300 *μ*L of 0.25 M HAuCl_4_·3H_2_O in 200 mL of DI water. The color of the mixture changes gradually from yellow to colorless within 30 min. The solution is then aged for 1 day before use. The growth of the gold shell is done in a step-wise manner as follows. Initially, 200 *μ*L of the prepared Fe_3_O_4_ NRs@SiO_2_-Au seed (0.67 mg/mL) is mixed with 1 mL growth solution, and then 25 *μ*L formaldehyde (3.7%) is added. Further additions of 1 mL growth solution and 25 uL formaldehyde are added at 2 min intervals until a total of 18 mL growth solution has been added. For each 4 mL of growth solution added, 50 *μ*L PVP (5 wt%, average M.W. 3500) is added before continuing the addition sequence. After the addition sequence is complete, 500 *μ*L PVP is added to the solution to stabilize the gold surface and prevent aggregation. During the reaction, the color of the solution gradually changes from nearly colorless to light pink, pink, red, purple, blue-green, and finally blue-shifting back to a purplish-blue. The mixture is incubated overnight, and excess PVP is removed by centrifugation and washing with water. The final product (PVP-modified Fe_3_O_4_ NRs@SiO_2_@Au shell) is concentrated into 1 mL of aqueous solution. According to our test, the reaction for the growth of gold shell can be scaled up by at most 50 times, if more samples are needed.

### 4.4. Magnetically Controlled Angle-Dependent Plasmonic Property Measurement

The extinction spectra of the nanocomposites are measured using a Varian Cary 500 double beam scanning UV/Vis/NIR spectrophotometer. CS_2_ is used as the solvent to eliminate the influence of water in the near-infrared region. The surface of Fe_3_O_4_ NRs@SiO_2_@Au shell is modified with alkane-thiol ligands to obtain the excellent dispersibility of the nanocomposites in CS_2_. Briefly, 10 mL of PVP-modified Fe_3_O_4_ NRs@SiO_2_@Au shell aqueous solution is dispersed in ethanol, then 0.25 mL of 1-octanethiol is added under stirring, and the mixture incubated overnight. The alkane-functionalized nanocomposites are then washed with ethanol and dispersed in CS_2_.

The sample is placed in a quartz cuvette with 1 cm path length. Linearly Y-polarized light (Figure 2(a)) was achieved by using three polarizers with operating wavelength ranges located at 400-700 nm, 600-1100 nm, and 1050-1700 nm. An external magnetic field is applied and actuated using a servo motor equipped with a permanent magnet. The distance between the magnet and the sample is fixed at 1.5 cm. For varying the angle *θ*, the polarization direction of the incident light is fixed, and the magnetic field direction is rotated in the XY plane.

### 4.5. FDTD Simulation of the Plasmonic Extinction

The finite-difference time-domain (FDTD) method is used to calculate the extinction spectra and associated electric field distributions by using a total-field scattered-field source (TFSF). The Fe_3_O_4_ @SiO_2_ NRs@Au shell employed for the FDTD simulations is composed of the iron oxide core with a length of 259 nm and width of 49 nm surrounded by a 45-nm thick silica coating and 36.5-nm thick Au shell. A nonuniform meshing grid with smallest grid size ≈ 1 nm is used. The refractive indices of gold and silica were adopted from Palik's Handbook of Optical Constants of Solids [33], while the refractive index data for Fe_3_O_4_ was adopted from Schlegel et al. [34]. The refractive index of surrounding CS_2_ is set to be 1.62.

### 4.6. Fabrication of Polymer Films Consisting of an Array of Nanocomposites with Select Orientations

Polymer films with select plasmon extinction values are prepared by fixing the nanocomposites in a matrix of PEGDA through photopolymerization. The PVP-modified nanocomposites were first functionalized with poly(ethylene glycol) methyl ether thiol (PEG-SH) through ligand-exchange to improve dispersibility in PEGDA. The nanocomposites made from 4 mL of the seed solution (scale up by 20 times of a typical synthesis) are first washed with ethanol and dispersed into 20 mL PEGDA solution. 0.5 g of the photoinitiator, phenylbis(2,4,6-trimethylbenzoyl)phosphine oxide (2.5 wt%), is added to the mixture which is then sonicated for a while to ensure complete dissolution of the photoinitiator. The mixture is sandwiched between one glass slide (at the bottom) and one quartz slide (on top) separated by a glass spacer of 0.1 cm in height. The orientation of the nanocomposites is selected by applying an external magnetic field; after 1 min, the solution is cured by exposing to the UV light source for 20 s. Film** 0**, Film** 1**, and Film** 2** are prepared using magnetic field directions of 0°, 45°, and 90° to the plane of the polymer film respectively. All the films are 2.5 cm *∗* 2.0 cm, with a thickness of 0.1 cm.

### 4.7. Design and Setup of the Homemade IRPECS

Diagram of the homemade IR-photoelectric coupling system (IRPECS) is shown in Figure 3(d). An infrared LED (THORLABS, LED1600L) with a working voltage of 1.05 V and current of 0.10 A is used in conjunction with a linear film polarizer (THORLABS, LPIREA100-C) to provide the linearly Y-polarized or Z-polarized incident light. An IR photodiode (THORLABS, FGA015) and a signal amplifier (DROK, AD620) with a bias voltage of 6.0 V are used to detect changes in the intensity of the incident light passing through the sample. The IR-LED and IR photodiode are fixed in place with a distance of 12 mm apart. A sample guide slot made of PDMS was used to maintain a constant distance between the sample and the detector as the various films are scanned. For testing of the liquid sample, a cuvette (0.5 cm path length) containing the sample of nanocomposites in PEGDA solution is placed between the light source and the photodiode and the voltage signal from the IRPECS is recorded by an oscilloscope (OWON, VDS1022I).

### 4.8. Information Encryption by Using Nanocomposites Film Array

Individual nanocomposite films (Films** 0, 1,** and** 2**) of 1.6 cm *∗* 0.8 cm in size and 0.1 cm thick are prepared as described above. Six of these films are arranged into a sequence (i.e.,** 012012**) and sandwiched between one large glass slide (at the bottom) and one large quartz slide (on top) separated by a 0.1 cm glass spacer. A pure PEGDA solution with 2.5 wt% of photoinitiator is then added to fill the remaining space between the sandwiched films. The sample is further cured under UV irradiation, and the final result is a larger film array having sequentially encoded extinction values.

### 4.9. Information Decryption by Using the IRPECS and the Identification System

To easily read the information from the encoded film array, we designed an identification system. The system consists of one programmed ARDUINO controller and a common anode LED (Chanzon, RGB). The anode of the LED was connected to the 3.3 V pin and the cathode to three different PWM pins of the ARDUINO. The voltage signal from the IRPECS is connected to GND and the ANALOG-IN pins of the ARDUINO. For Films** 0, 1,** and** 2**, the emission colors were programmed to be red, green and purple (red + blue), respectively. For decrypting the encoded film array, the film array is placed into the guide slot of the IRPECS and gradually scanned from left to right, so that each encoded subunit (Films** 0**-**2**) can be measured. The film is scanned twice, once with Y-polarized light and then with Z-polarized light.

### 4.10. Design and Setup of a Novel Magnetic-Field-Direction Sensor

The magnetic-field-direction sensor is demonstrated by a combination of the IRPECS and a liquid sample of nanocomposites. Briefly, a sample of nanocomposites dispersed in PEGDA solution in a cuvette is placed between the light source and the photodiode of the IRPECS which reads out a voltage corresponding to the orientation of the nanocomposites, effectively indicating the magnetic field direction. Additionally, the sensor is connected to an ARDUINO to actuate a MeArm base servo motor. The voltage signal from the IRPECS is connected to GND and ANALOG-IN pins of the ARDUINO. The MeArm base servo motor was connected to 5.0V GND and PWM pins of the ARDUINO. The MeArm is set to rotate around the motor base axis between 0° to 90° (angle *α*) which corresponds to the minimum and maximum of the IRPECS output voltage, respectively. Linearly Y-polarized light is used as the incident light, and the magnetic field direction is varied in the XY plane between 0° to 90° by manually rotating a permanent external magnet approximately 1.5 cm away from the cuvette.

## Data Availability

All data needed to evaluate the conclusions in the paper are present in the paper and the Supplementary Materials. Additional data related to this paper may be requested from the authors.

## Supplementary Materials

Supplementary Materials Figure S1: XRD patterns of *β*-FeOOH nanorods before and after reduction. Figure S2: the enlarged low-field curve of the magnetic hysteresis loop of Fe_3_O_4_ NRs@SiO_2_. Figure S3: UV-Vis spectra and TEM images of products during the seeded growth process. Figure S4: extinction spectra of magnetic nanorod with and without an external magnetic field. Figure S5: extinction spectra of CS_2_ and nanocomposites when *θ* is 90°. Figure S6: characterization of the IR light source and photodiode used in the IRPECS. Figure S7: diagram of the IRPECS and identification system. Figure S8: 27 possible combinations of information encryption based on a 3-column film. Figure S9: *θ* angle and voltage plotted over time. Movie S1: information decryption when using Y-polarized light. Movie S2: information decryption when using Z-polarized light. Movie S3: 27 combinations of information encryption based on a 3-column film. Movie S4: setup for controlling the rotation of the magnet using a servomotor. Movie S5: an actuator system based on the magnetic-filed-direction sensor.
