# Supplementary material for: Anisotropically Shaped Magnetic/Plasmonic Nanocomposites for Information Encryption and Magnetic-Field-Direction Sensing
Source: Research (Wash D C). 2018 Aug 30;2018:7527825. doi: 10.1155/2018/7527825 (PMC6750074; doi:10.1155/2018/7527825)
Supplement: Supplementary Materials — Figure S1: XRD patterns of β-FeOOH nanorods before and after reduction. Figure S2: the enlarged low-field curve of the magnetic hysteresis loop of Fe3O4 NRs@SiO2. Figure S3: UV-Vis spectra and TEM images of products during the seeded growth process. Figure S4: extinction spectra of magnetic nanorod with and without an external magnetic field. Figure S5: extinction spectra of CS2 and nanocomposites when θ is 90°. Figure S6: characterization of the IR light source and photodiode used in the IRPECS. Figure S7: diagram of the IRPECS and identification system. Figure S8: 27 possible combinations of information encryption based on a 3-column film. Figure S9: θ angle and voltage plotted over time. Movie S1: information decryption when using Y-polarized light. Movie S2: information decryption when using Z-polarized light. Movie S3: 27 combinations of information encryption based on a 3-column film. Movie S4: setup for controlling the rotation of the magnet using a servomotor. Movie S5: an actuator system based on the magnetic-filed-direction sensor. [file 7527825.f1.zip › 7527825.f1/Wang-supporting-revised.docx]

Supplementary Materials for

**Anisotropically Shaped Magnetic/Plasmonic Nanocomposites for Information Encryption and Magnetic-Field-Direction Sensing**

Xiaojing Wang^1,2^, Ji Feng^1^, Huakang Yu^3^, Yue Jin^1^, Andrew Davidson^1^, Zhiyuan Li^3^, and Yadong Yin^1, 2^*

^1^Department of Chemistry, University of California, Riverside, CA 92521, USA.

^2^Materials Science and Engineering Program, University of California, Riverside, CA 92521, USA.

^3^School of Physics and Optoelectronics, South China University of Technology, Guangzhou 510641, China

*e-mail: yadong.yin@ucr.edu

**The Supplementary Materials document includes:**

- fig. S1. XRD patterns of β-FeOOH nanorods before and after reduction.
- fig. S2. The enlarged low-field curve of the magnetic hysteresis loop of Fe_3_O_4_ NRs@SiO_2_.
- fig. S3. UV-Vis spectra and TEM images of products during the seeded growth process.
- fig. S4. Extinction spectra of magnetic nanorod with and without an external magnetic field.
- fig. S5. Extinction spectra of CS_2_ and nanocomposites when θ is 90°.
- fig. S6. Characterization of the IR light source and photodiode used in the IRPECS.
- fig. S7. Diagram of the IRPECS and identification system.
- fig. S8. 27 possible combinations of information encryption based on a 3-column film.
- fig. S9. θ angle and voltage plotted over time.
- Legends for movies S1 to S5

**Other Supplementary Material for this manuscript includes the following:**

- Movie S1. Information decryption when using Y-polarized light.
- Movie S2. Information decryption when using Z-polarized light.
- Movie S3. 27 combinations of information encryption based on a 3-column film.
- Movie S4. Setup for controlling the rotation of the magnet using a servomotor.
- Movie S5. An actuator system based on the magnetic-filed-direction sensor.

**Supplementary Materials**


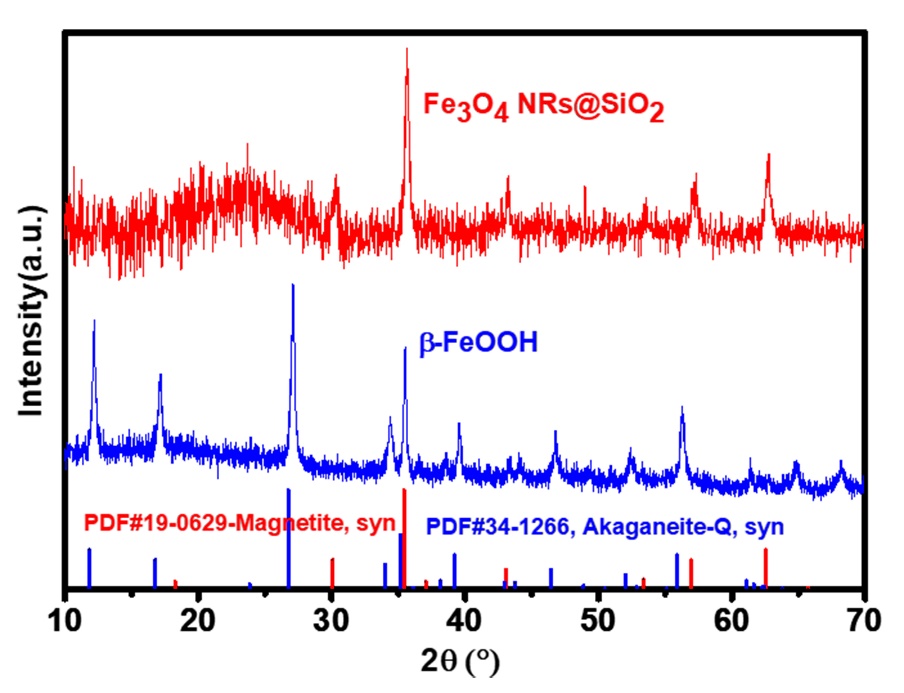


**fig. S1. XRD patterns of β-FeOOH nanorods before and after reduction.** Before the forming gas reduction, the phase of the nanorods was determined to be akaganéite (blue line). After reduction, the phase becomes magnetite (red line).


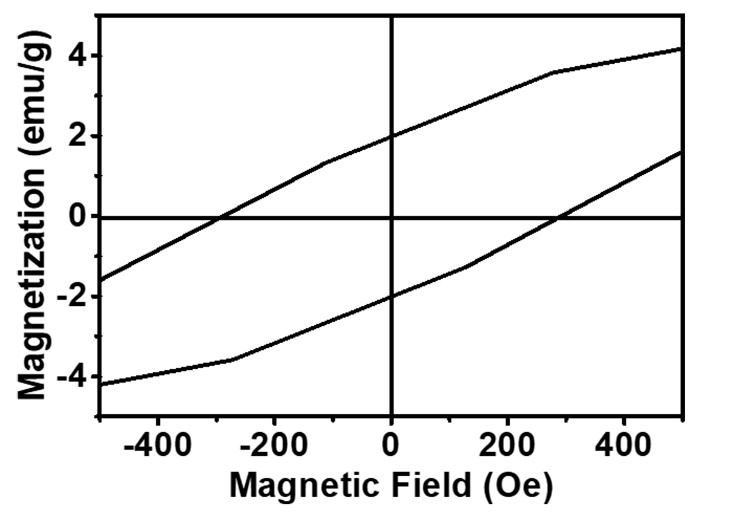


**fig. S2. The enlarged low-field curve of the magnetic hysteresis loop of Fe_3_O_4_ NRs@SiO_2_.** The coercivity was measured to be 290 G, which confirms the ferromagnetic property of the reduced samples.


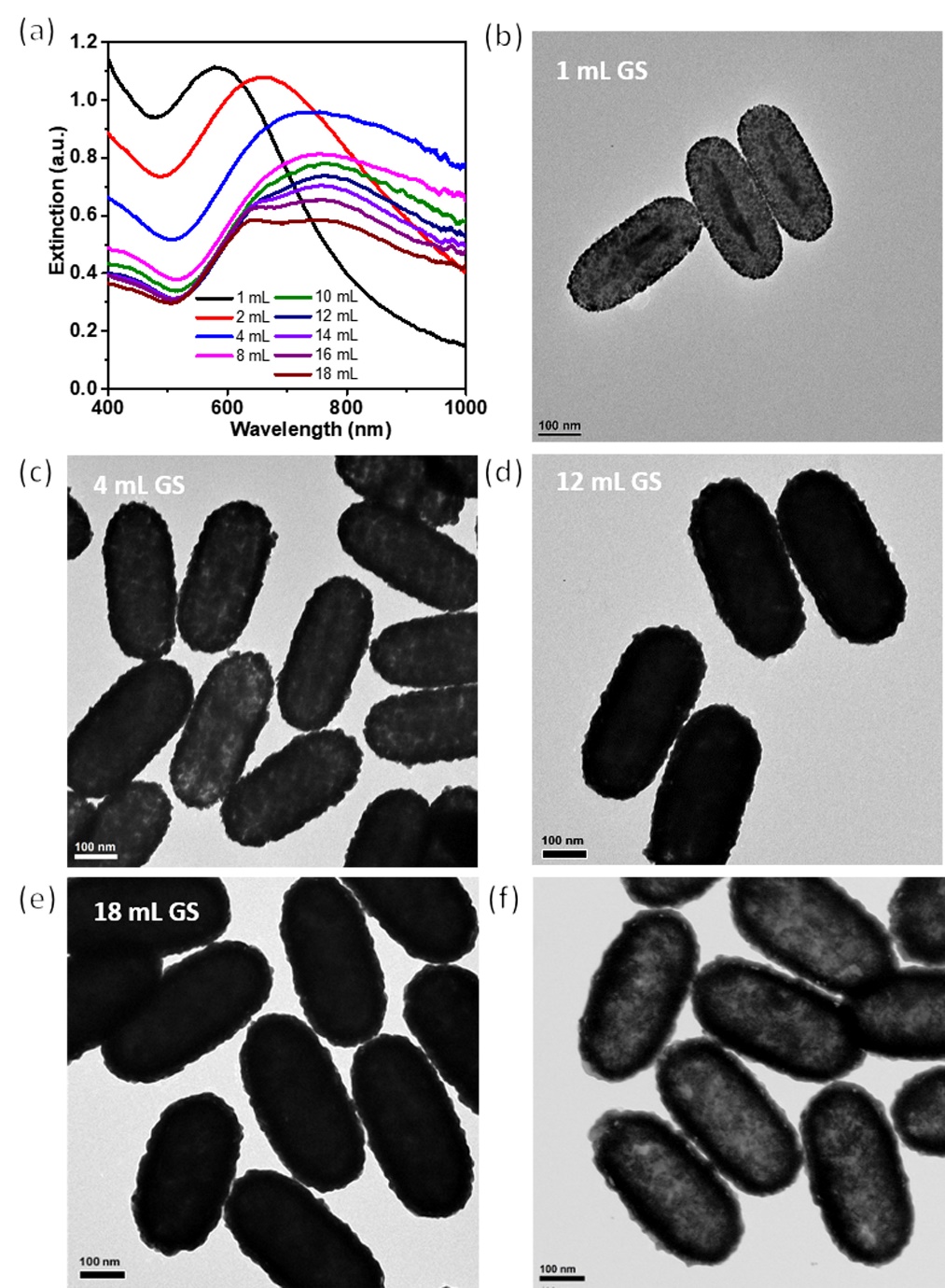


**fig. S3.** **UV-Vis spectra and TEM images of products during the seeded growth process.** (**a**) UV-Vis spectra measured during the seeded growth process with different amount of the gold growth solution. (**b-e**) TEM images of the nanocomposites when the amount of the gold growth solution is 1, 4, 12, 18 mL, respectively. **(f)** The Au nanoshells obtained after removing the Fe_3_O_4_/SiO_2_ core from the 18-mL sample. The contrast of the image was manipulated to reveal the shell structure.


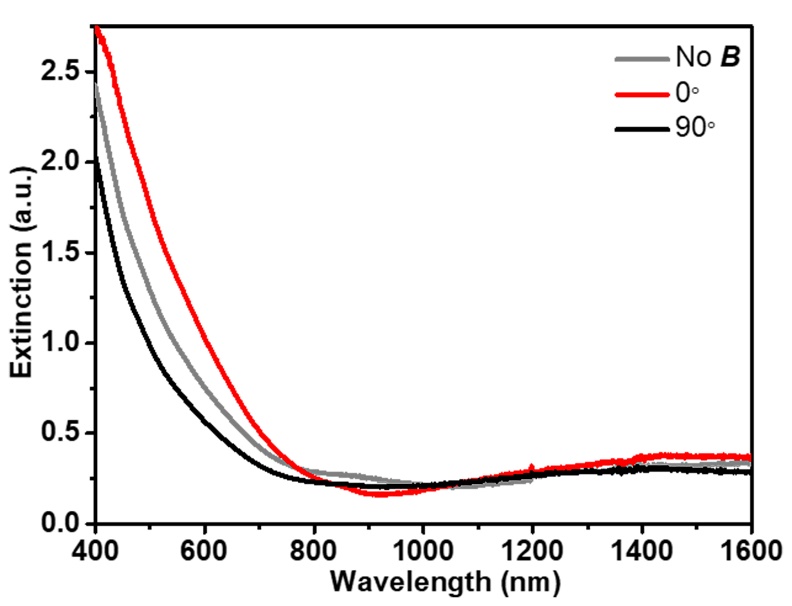


**fig. S4.** **Extinction spectra of magnetic nanorod with and without an external magnetic field.** Extinction spectra of the Fe_3_O_4_@SiO_2_ NRs in PEGDA solution with θ of 90 ° (black line) and 0° (red line), and without applying the external magnetic field (grey line), respectively.


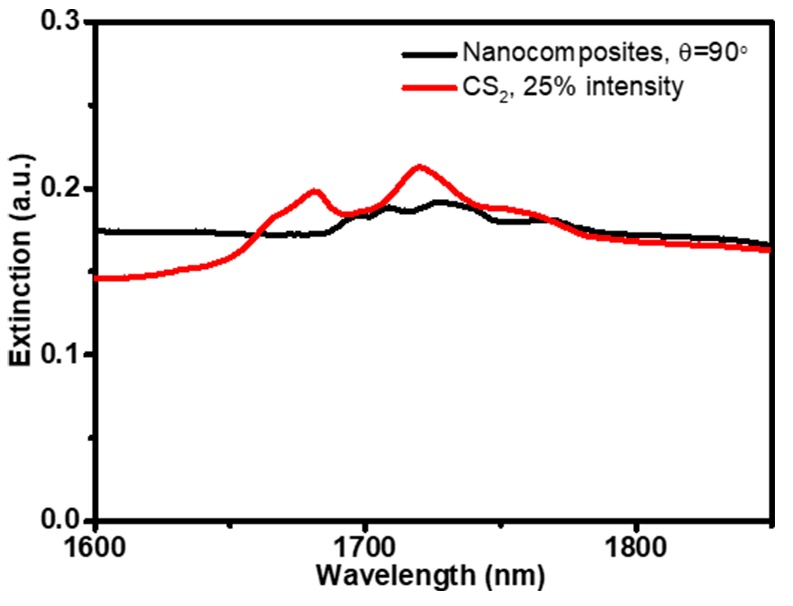


**fig. S5. Extinction spectra of CS_2_ and nanocomposites when θ is 90°.** The small peaks located in the range of 1650 – 1800 nm are verified to be CS_2_ solvent employed in the measurement.


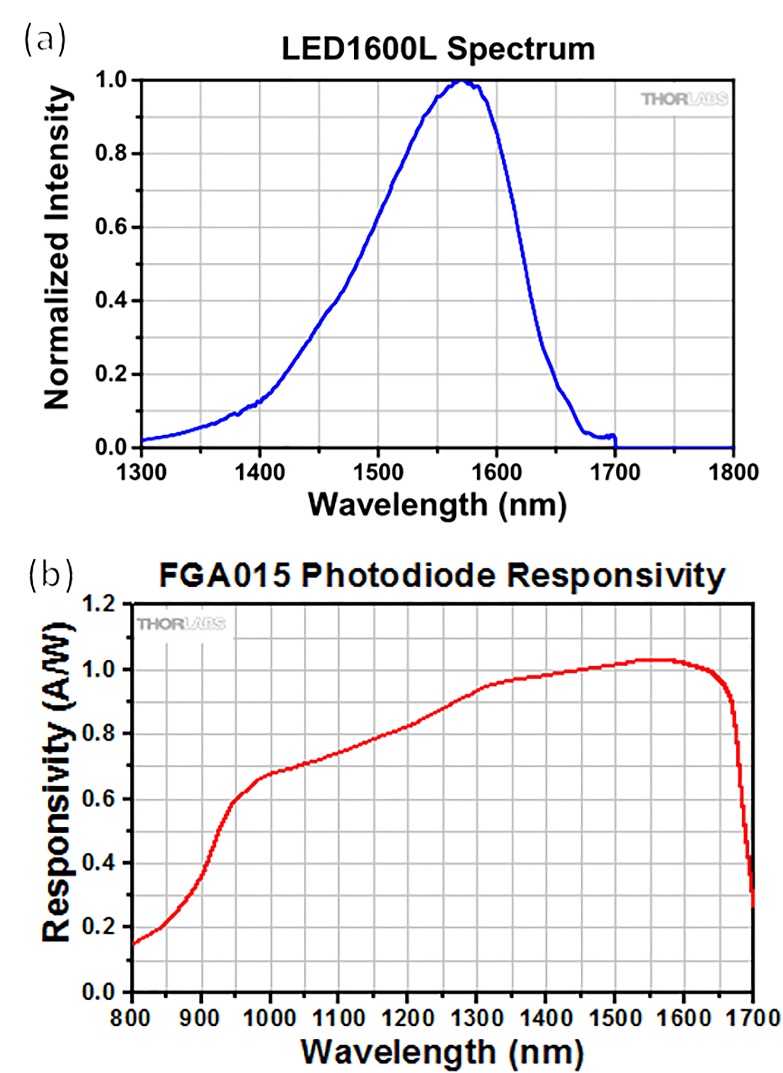


**fig. S6. Characterization of the IR light source and photodiode used in the IRPECS.** Spectrum of the IR light source (**a**) and photodiode responsivity profile (**b**). These two figures were downloaded from the website of THORLABS; the links are listed below. <https://www.thorlabs.com/thorproduct.cfm?partnumber=LED1600L>

<https://www.thorlabs.com/thorproduct.cfm?partnumber=FGA015>


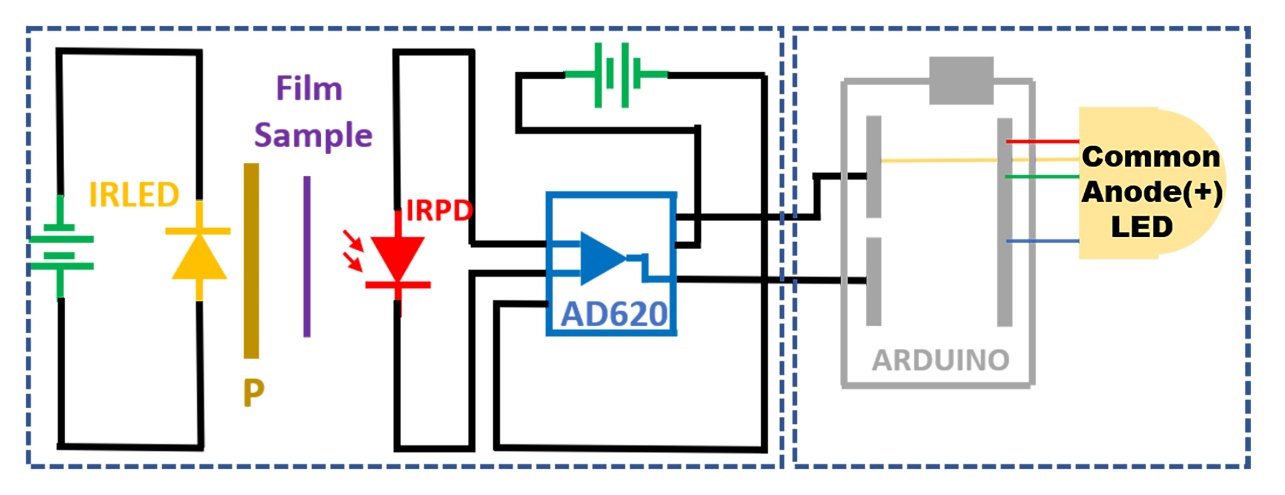


**fig. S7.** **Diagram of the IRPECS and identification system.** Diagram of the homemade IR photoelectric coupling system (IRPECS, left part) combined with the identification system (right part), which is a common anode LED that connected to the IRPECS through ARDUINO.


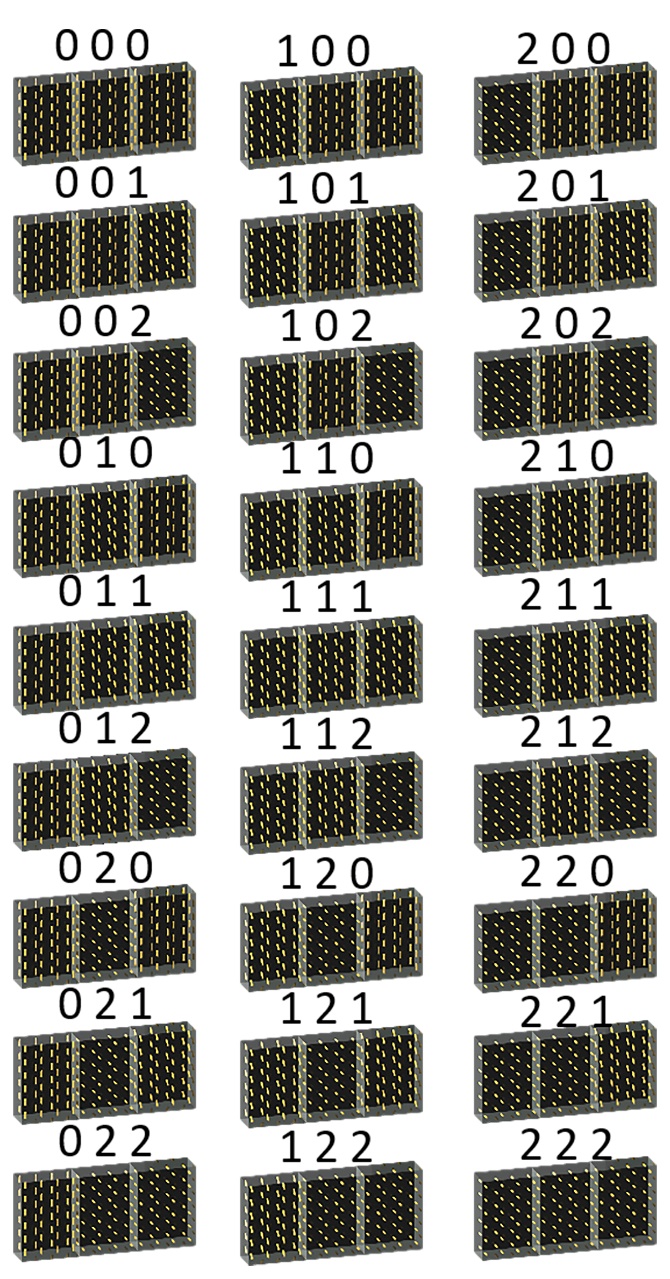


**fig. S8. 27 possible combinations of information encryption based on a 3-column film.** Under the Y-polarized light, all the 27 films show a “222” reading; but when decrypted using Z-polarized light, they will show different readings, which listed above the scheme of the film.


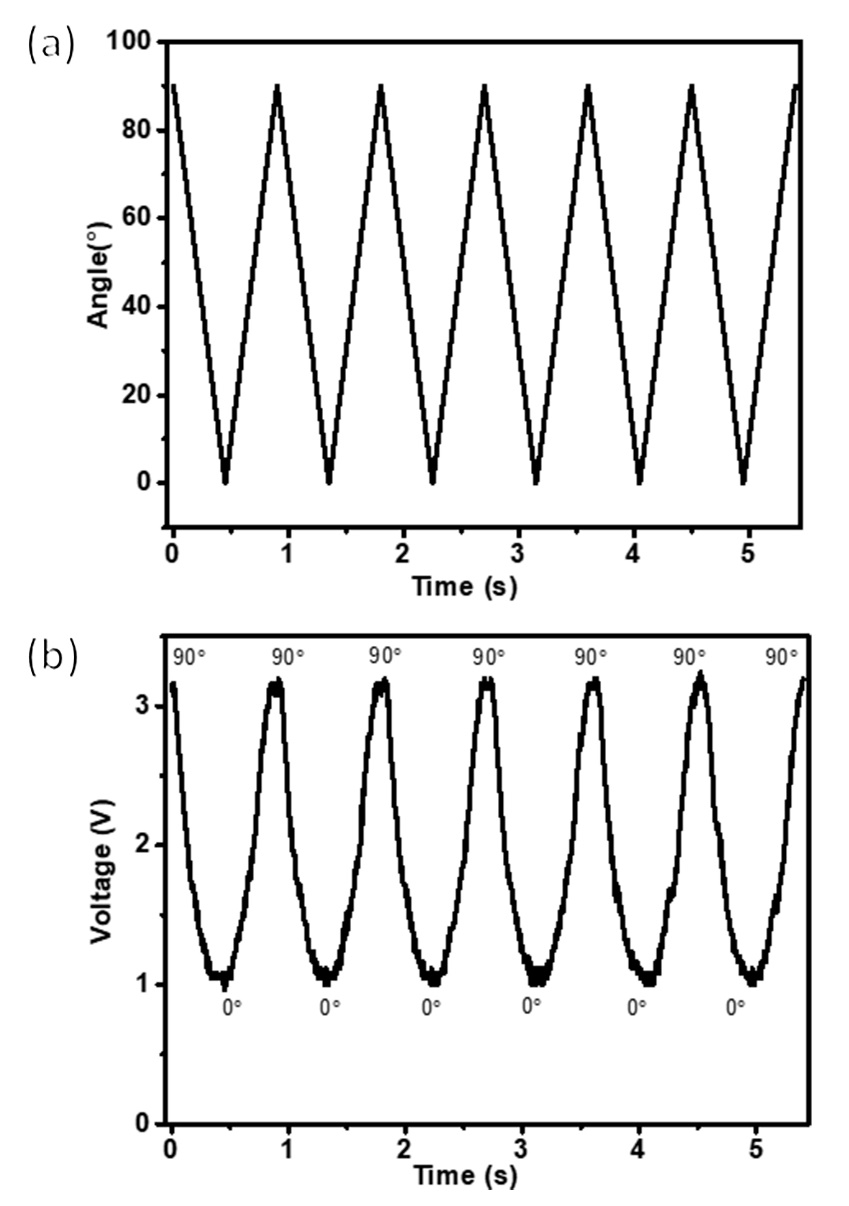


**fig. S9.** **θ angle and voltage plotted over time.** θ angle (**a**) and voltage (**b**) plotted over time when using a servo motor which equipped with a permanent magnet to apply the external magnetic field. The rotation rate of the servo motor is 10° per 50 ms. The voltage was measured by using the homemade IR photoelectric coupling system (Fig. 3d for circuit diagram). Insets in (**b**) are the corresponding θ angles.

**Movie S1.** **Information decryption when using Y-polarized light.** The LED readout shows the same purple color for all six subunits of the encrypted film array when measured using Y-polarized light. Since the nanorods are aligned in the XZ plane, and the angle between the longitudinal axis of the nanocomposites and Y-axis is kept at 90°, there is no output voltage change.

**Movie S2.** **Information decryption when using Z-polarized light.** When the Z-polarized light is used, the three subunits of the film array cause the LED to vary in color among purple, green, and red. Based on the LED color and a decryption key, the readout can be recorded as **012012**. Since the nanorods are aligned in the XZ plane, and the angle between the longitudinal axis of the nanocomposites and Z-axis is varying among 0°, 45°, and 90°, the output voltage will change accordingly.

**Movie S3.** **27 combinations of information encryption based on a 3-column film.** Under the Y-polarized light, all the 27 films show a “**222**” reading; but when decrypted using Z-polarized light, they will have different readings, as shown in the movie.

**Movie S4.** **Setup for controlling the rotation of the magnet using a servomotor.** A servo motor equipped with a permanent magnet provides the external magnetic field, and the motor was programmed to rotate continuously at a different speed. The movie shows the rotation of the motor at a speed of 10° per 50 ms and 10° per 500 ms, respectively.

**Movie S5.** **An actuator system based on the magnetic-filed-direction sensor.** As shown in the movie, the magnetic field direction sensor is connected to an ARDUINO to actuate a MeArm base servo motor. The MeArm is set to rotate around the motor base axis between 0° to 90° which corresponds to the minimum and maximum of the IRPECS output voltage respectively. When changing θ by manually rotating the permanent external magnet placed beside the cuvette, the robotic arm can respond and rotate accordingly
